# Composite pheochromocytoma of the adrenal gland: a case series

**DOI:** 10.1186/s13104-015-1233-6

**Published:** 2015-06-24

**Authors:** Yohei Shida, Tsukasa Igawa, Kuniko Abe, Tomoaki Hakariya, Kousuke Takehara, Toru Onita, Hideki Sakai

**Affiliations:** Department of Nephro-Urology, Nagasaki University Graduate School of Biomedical Sciences, 1-7-1 Sakamoto, Nagasaki, 852-8501 Japan; Department of Pathology, Nagasaki University Graduate School of Biomedical Sciences, 1-7-1 Sakamoto, Nagasaki, 852-8501 Japan

**Keywords:** Composite pheochromocytoma, Ganglioneuroblastoma, Ganglioneuroma, Adrenal gland, Hemodialysis, Ki67

## Abstract

**Background:**

Composite pheochromocytoma is a rare pathological condition characterized by elements of both pheochromocytoma and neurogenic tumors. However, detailed clinical outcomes of this tumor have not been fully shown. From 2007 to 2013, we experienced three cases of adrenal composite pheochromocytoma. In this report, we investigate the clinicopathological features of these three cases of composite pheochromocytoma and compare them with previously reported cases.

**Case presentations:**

Cases 1 and 2 were a 29-year-old Japanese woman and a 59-year-old Japanese man, respectively. They underwent laparoscopic left adrenalectomy, and pathological examination revealed composite pheochromocytoma–ganglioneuroma. Case 3 was a 53-year-old Japanese man who had been receiving hemodialysis for 17 years. He underwent laparoscopic right adrenalectomy, and pathological examination revealed composite pheochromocytoma–ganglioneuroblastoma. Although the Ki67-positive rates varied from 1.0 to 6.2% among the three cases, no clinical recurrences occurred. Despite the relatively high rate of Ki67 positivity, complete tumor resection resulted in favorable clinical outcomes.

**Conclusion:**

We experienced three cases of adrenal composite pheochromocytoma. Although the clinical findings and treatment outcomes of composite pheochromocytoma were similar to those of ordinary pheochromocytoma, further studies of the biological behavior and genetic profiles of composite pheochromocytoma are necessary to achieve a better understanding of this tumor.

## Background

Ordinary pheochromocytoma is a neuroendocrine tumor derived from the adrenal medulla. It consists of polygonal and spindle cells arranged in alveolar, trabecular, or solid patterns and often exhibits a typical Zellballen pattern. Composite pheochromocytoma is a rare tumor composed histologically of pheochromocytoma and other neurogenic tumor components such as neuroblastoma, ganglioneuroblastoma, ganglioneuroma, peripheral nerve sheath tumor, or other types of neuroendocrine carcinoma [1]. The frequency of composite adrenal tumors reportedly ranges from <3% of all adrenal gland neoplasms to 1–9% of pheochromocytomas. Composite pheochromocytoma is relatively rare among composite adrenal tumors, accounting for only 3% of pheochromocytomas [1, 2]. We experienced three cases of adrenal composite pheochromocytoma from 2007 to 2013 (Table 1). Two of these were compound tumors comprising pheochromocytoma and ganglioneuroma, and the other was a tumor comprising pheochromocytoma and ganglioneuroblastoma. We herein report these three cases to help achieve a better understanding of composite pheochromocytoma.

**Table 1:** Clinicopathological findings of three composite pheochromocytomas

Case	Sex	Clinical manifestation	Tumor size (cm)	Morphologic feature	Ki67 index^a^ (%)
1	F/29	Palpitation	4.5	Ganglioneuroma	4.3
2	M/59	Asymptomatic hematuria	5.5	Ganglioneuroma	1.0
3	M/53	Asymptomatic hematuria, hypertension	3	Ganglioneuroblastoma	6.2

^a^Five areas of greater Ki67 expression intensity (hot spots) were selected. At least 500 cells were counted, and the ratio between the number of cells with a positively stained nucleus and the total number of cells was calculated.

## Case presentations

### Case 1

**Figure 1:**
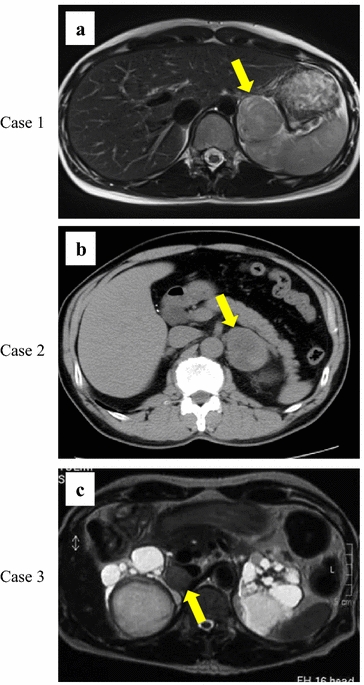
Computed tomography or magnetic resonance imaging of three composite pheochromocytomas. **a** Magnetic resonance imaging revealing a heterogeneous left adrenal mass (indicated by *arrowhead*) with slightly high intensity on T2-weighted images. **b** Computed tomography scan showing a left adrenal mass (indicated by *arrowhead*). **c** Magnetic resonance imaging showing acquired cystic kidney disease and a right adrenal mass (indicated by *arrowhead*).

**Figure 2:**
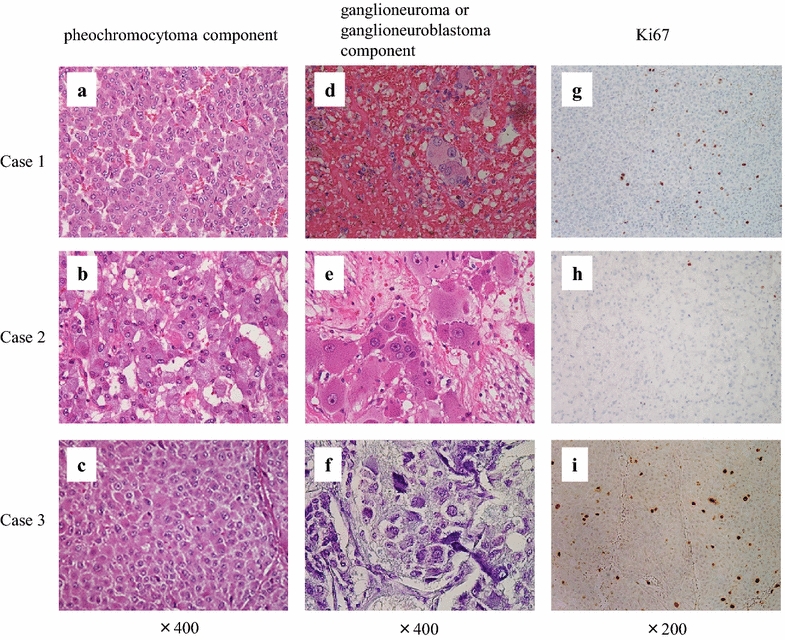
Microscopic findings of resected tumors. *Left lanes* (**a**–**c**) pheochromocytoma component (hematoxylin and eosin staining, ×400). *Middle lanes* (**d**–**f**) ganglioneuroma or ganglioneuroblastoma components. **d**, **e** Ganglioneuroma component (hematoxylin and eosin staining, ×400). **f** Ganglioneuroblastoma component (Nissl staining, ×400). *Right lanes* (**g**–**i**) positive immunohistochemical staining of the pheochromocytoma component with Ki67 (×200).

A 29-year-old Japanese woman presented with paroxysmal palpitation and headache. She had no history of hypertension. Blood catecholamine analysis during the palpitation revealed epinephrine, norepinephrine, and dopamine levels of 417 pg/ml (reference range, 0–100 pg/ml), 2,665 pg/ml (reference range, 100–450 pg/ml), and 16 pg/ml (reference range, 0–60 pg/ml), respectively. Magnetic resonance imaging revealed a heterogeneous left adrenal mass with slightly high intensity on T2-weighted images (Figure 1a). ^123^I-meta-iodobenzylguanidine (MIBG) scintigraphy showed a high level of accumulation in the left adrenal tumor. After diagnosis of left adrenal pheochromocytoma, the patient underwent laparoscopic left adrenalectomy. Pathological findings showed a composite pheochromocytoma-ganglioneuroma in which 4.3% of cells were Ki67-positive (Figure 2a, d, g). She was followed closely after surgery. Despite the high number of Ki67-positive cells, no recurrence was observed for 2 years.

### Case 2

A 59-year-old Japanese man presented with asymptomatic macrohematuria. He had a history of angina pectoris and took a daily dose of aspirin. Abdominal ultrasonography showed no evidence of a renal or bladder tumor, and cystoscopy showed no evidence of a bladder tumor. Computed tomography revealed a 5.5-cm left adrenal mass (Figure 1b). Blood catecholamine analysis revealed epinephrine, norepinephrine, and dopamine levels of 136 pg/ml (reference range, 0–100 pg/ml), 458 pg/ml (reference range, 100–450 pg/ml), and 15 pg/ml (reference range, 0–60 pg/ml), respectively. ^123^I-MIBG scintigraphy showed a high level of accumulation in the left adrenal gland. After diagnosis of a left adrenal pheochromocytoma, the patient underwent laparoscopic left adrenalectomy. Pathological examination revealed a composite pheochromocytoma–ganglioneuroma with focal Ki67-positive cells (1%) (Figure 2b, e, h).

### Case 3

A 53-year-old Japanese man presented with asymptomatic macrohematuria. He had undergone hemodialysis for 17 years and had a history of hypertension. Cystoscopy showed no evidence of a bladder tumor. Abdominal magnetic resonance imaging showed acquired cystic kidney disease and a 3-cm right adrenal mass (Figure 1c). Serum catecholamine analysis revealed an epinephrine level of 50 pg/ml (reference range, 0–100 pg/ml), norepinephrine level of 3,801 pg/ml (reference range, 100–450 pg/ml), and dopamine level of 90 pg/ml (reference range, 0–60 pg/ml). ^123^I-MIBG scintigraphy revealed strong accumulation in the right adrenal gland. After diagnosis of a right adrenal pheochromocytoma, the patient underwent laparoscopic right adrenalectomy. Pathological examination revealed a composite pheochromocytoma–ganglioneuroblastoma with a 6.2% rate of Ki67-positive cells (Figure 2c, f, i). Despite the unfavorable tumor histology, he remained tumor-free for 6 years after the surgery.

In the present report, two patients showed asymptomatic hematuria. However, cystoscopy showed no evidence of a bladder tumor. The ratio of eventual coexistence of hematuria and composite pheochromocytoma was unknown, although we reviewed previous reports.

## Discussion

Tumors derived from the adrenal medulla are classified as either pheochromocytomas or neuroblastic tumors. Composite pheochromocytoma is a rare pathological entity that is composed of pheochromocytoma and other histological components, most frequently neuroblastic elements. Neuroblastic tumors such as neuroblastoma, ganglioneuroblastoma, and ganglioneuroma are tumors of the sympathetic nervous system that arise from primitive sympathogonia. They are usually observed in the neck, posterior mediastinum, adrenal gland, retroperitoneum, and pelvis [3]. The diagnosis of a composite tumor is currently based only on the histopathological findings. Shawa et al. [4] reported that pheochromocytoma–ganglioneuroma composites are clinically and radiologically indistinguishable from pheochromocytomas and need to be managed similarly.

Pheochromocytoma was often termed “the 10% tumor” at the end of the 20th century. However, pheochromocytoma is not described this way any longer. Until 2002, 10% of all pheochromocytomas were considered to be hereditary. However, among 271 patients who presented with nonsyndromic pheochromocytoma and without a family history of the disease, 66 (24%) were found to have a hereditary predisposition [5]. In another study, Amar et al. [6] reported that 86 (27.4%) of 314 patients had a hereditary pheochromocytoma or functional paraganglioma.

Several genes are known to play an important role in the pathogenesis of pheochromocytoma, including the *RET* proto-oncogene, von Hippel–Lindau disease tumor suppressor (*VHL*), neurofibromatosis type 1 tumor suppressor (*NF1*), genes encoding the succinate dehydrogenase (SDH) complex subunits, *TMEM127*, and MYC-associated factor X (*MAX*) [7]. Neumann et al. [5] suggested that routine analysis for mutations of the *RET*, *VHL*, *SDHD*, and *SDHB* genes in patients with apparently sporadic presentation of pheochromocytoma should be considered as the clinical standard of care. However, whether these genetic alterations are also associated with composite pheochromocytoma remains unclear.

Kikuchi et al. [8] reported that the histological transformation from pheochromocytoma to a composite tumor might occur over a long clinical course. They suggested that age-associated changes to the microenvironment might influence the differentiation of pheochromocytoma cells into neuronal cells. Therefore, it is possible that the changes to the microenvironment caused by chronic renal failure influence the pathogenesis of ordinary/composite pheochromocytoma, as with Case 3 in the present report. Nevertheless, the precise mechanism of histopathological transformation is unclear. Approximately 71% of composite pheochromocytomas coexist with ganglioneuroma. These tumors occur with equal frequency in males and females, and most patients are aged 40–60 years [9].

Ganglioneuroma is the most common benign tumor among neuroblastic tumors and is composed of gangliocytes and mature stroma. In contrast, ganglioneuroblastoma is composed of mature gangliocytes and immature neuroblasts [3]. It exhibits malignant potential and occurs most commonly in infants and young children; it presents only rarely after 10 years of age [2]. Composite pheochromocytoma–ganglioneuroblastoma is an extremely rare tumor [10, 11], particularly in patients undergoing hemodialysis, such as Case 3 in the current study. Fujiwara et al. [10] reviewed seven patients with composite pheochromocytoma–ganglioneuroblastoma. Two patients died of metastasis from a malignant pheochromocytoma component. Five patients with localized tumors underwent complete resection with no adjuvant chemotherapy or radiation; all of these patients were alive at the time of publication.

In recent years, the estimation of proliferative activities using Ki67 immunostaining has become an important tool when assessing endocrine tumors. Tavangar et al. [12] indicated that pheochromocytomas with malignant behavior usually contain >5% Ki67-positive nuclei. In the present cases, the rate of Ki67-positive cells was 6.2, 4.3, and 1.0%, respectively, and all patients remained free of tumor recurrence for up to 6 years postsurgery without adjuvant chemotherapy or radiation. Kimura et al. [13] reported a scoring scale based on six factors: histological pattern, cellularity, coagulation necrosis, vascular/capsular invasion, Ki67 immunoreactivity, and types of catecholamine produced. This grading system for adrenal pheochromocytoma and extra-adrenal sympathetic paraganglioma might be useful for predicting patients’ prognosis. However, the clinicopathological indicators necessary to accurately predict the prognosis in patients with composite pheochromocytoma have not been fully established. Therefore, in cases with a high probability of malignancy, adequate and accurate postoperative follow-up is required [14].

## Consent

Written informed consent was obtained from all patients for the publication of this case report and any accompanying images. A copy of the written consent is available for review by the Editor-in-Chief of this journal.

## Abbreviations

^123^I-MIBG: ^123^I-meta-iodobenzylguanidine
SDG: succinate dehydrogenase
*VHL*: von Hippel–Lindau disease tumor suppressor
*MAX*: MYC-associated factor X
